# Patient outcomes from lung cancer and diabetes mellitus: a matched case–control study

**DOI:** 10.4155/fsoa-2017-0081

**Published:** 2017-09-26

**Authors:** Nina J Karlin, Shailja B Amin, Matthew R Buras, Heidi E Kosiorek, Patricia M Verona, Curtiss B Cook

**Affiliations:** 1 Division of Hematology & Medical Oncology, Mayo Clinic Hospital, Phoenix, AZ, USA; 2 Department of Information Technology, Mayo Clinic Hospital, Phoenix, AZ, USA; 3 Department of Biostatistics, Mayo Clinic, Scottsdale, AZ, USA; 4 Division of Endocrinology, Mayo Clinic, Scottsdale, AZ, USA; 5 Division of Preventive, Occupational & Aerospace Medicine, Mayo Clinic, Scottsdale, AZ, USA

**Keywords:** diabetes, lung, malignancy, outcomes, survival

## Abstract

**Aim:**

This case–control study examined the impact of diabetes mellitus (DM) on survival in lung cancer patients and lung cancer on glycemic control in DM.

**Materials & methods:**

Patients with a new lung cancer diagnosis and DM (n = 124) were matched to 124 lung cancer patients without DM. Laboratory results and DM and cancer therapies were obtained from electronic records.

**Results:**

Five-year overall survival for lung cancer patients with and without DM was 20 versus 29% (p = .12). Glycemic control among DM patients did not change significantly with time.

**Conclusion:**

DM does not cause adverse impact on lung cancer survival. Lung cancer does not affect glycemic control.

## Lay abstract

The objective of this study was to identify the effect of diabetes mellitus (DM) on survival of lung cancer patients and to determine whether lung cancer and its treatment affect glycemic control. From an institutional cancer registry, 248 lung cancer patients were identified and categorized into two groups: those with lung cancer and DM (n = 124), and those with lung cancer but without DM (n = 124). The groups were matched by age and year of lung cancer diagnosis. Results indicated that DM does not adversely impact survival, and that lung cancer and its treatment do not affect control of DM.

Lung cancer is the second most common malignancy in men and women in USA, and is the leading cause of cancer-related death worldwide [1]. Most patients present with advanced disease, and the 5-year survival rate has stayed at 18% [2]. Concurrently, the prevalence of diabetes mellitus (DM) has continued to increase, with 9.3% of the US population having this diagnosis [3]. Projections cite that globally 439 million adults will have DM by 2030. Given the high prevalence of both lung cancer and DM, there will undoubtedly be scenarios where patients will present to their healthcare providers with both diagnoses and will require simultaneous DM and lung cancer management.

The association between DM and lung cancer risk continues to be controversial, with some studies reporting an increased risk of lung cancer with DM and others reporting a decreased risk [4]. As with many other types of malignancies that coexist with DM, the risk factors overlap, such as central adiposity and chronic inflammation [5–11]. Although total BMI is inversely related to lung cancer risk, waist circumference with central adiposity is positively associated with risk [5–8]. Outcome data are not consistent on survival of patients with both lung cancer and DM [12]. A recent study by Imai *et al*. [13] concluded that presence of DM at the time of lung cancer diagnosis was predictive of a negative outcome. In a meta-analysis, Zhu *et al*. [14] concluded that DM was associated with worse overall survival (OS) among lung cancer patients. However, some investigators have reported that patients with lung cancer and DM have an increased survival compared with those who have lung cancer but not DM. In addition, some data suggest that DM has a protective effect against metastasis in patients with non-small-cell lung cancer. However, few data are available regarding lung cancer effect on DM therapy or metabolic control in lung cancer patients with DM.

Given the potential individual and economic impacts of coexisting DM and lung cancer, understanding of their interactions is important to better inform providers on caring for these patients longitudinally. The authors have systematically analyzed data from their outpatient oncology practice with regard to how DM might affect cancer survival and how the presence of a solid organ malignancy might impact glycemic control or alter hyperglycemic therapy for patients with DM [15–19]. A prior analysis suggested no relationship between DM and lung cancer survival, but many variables were not available that might have affected conclusions [15]. Therefore, detailed patient data on DM and lung cancer variables were collected to analyze whether DM affected OS and progression-free survival (PFS), and investigate whether lung cancer and its treatment influenced metabolic control among the patients with DM.

## Methods

### Case selection

Institutional review board approval was obtained for this retrospective, case–control study. Similar to prior reports, health records of patients with newly diagnosed lung cancer who were seen in the practice from 1 January 2007 to 31 December 2015 were obtained from the institutional cancer registry. Patient data were collected on age at lung cancer diagnosis, diagnosis date, race/ethnicity and grade or stage of tumor. These data were cross-referenced against a list of all patients seen during that period who had a diagnosis of DM (*International Classification of Diseases, Ninth Revision*, diagnostic code 250.00) to categorize patients with lung cancer by DM status (with or without DM). Full or partial treatment at another institution or presence of another primary cancer, or both, were used as exclusion criteria. From this dataset, patients with lung cancer and DM were matched, at a 1:1 ratio using the Greedy algorithm [20], to control patients with lung cancer but no DM. Variables included in the matching algorithm were age, sex and year of diagnosis. Year of diagnosis was used as a matching variable to have a similar amount of follow-up available for patients with DM as for patients without DM.

Glucose and hemoglobin A_1c_ (HbA_1c_) values were derived from the laboratory information system. Health records were reviewed for additional detailed information about cancer stage, type, risk factors (smoking), type of lung cancer treatment received (e.g., surgery, chemotherapy, radiation therapy, immunotherapy and targeted therapy) and DM-related data (e.g., date of DM diagnosis, type of diabetic therapy and diabetic complications).

### Statistical analysis

Lung cancer cases with and without DM were compared for patient characteristics and clinical variables. Continuous variables were compared using paired *t* tests; categorical variables were compared using the McNemar test or Bowker test for symmetry. HbA_1c_ levels during the first year after lung cancer diagnosis were evaluated with a linear mixed model in DM cases only (HbA_1c_ values were unavailable for most patients without DM). Time (days) was considered a fixed effect, and an individual-specific random effect was included. A similar approach was used for modeling glucose values during that year. Fixed effects included days, case or control designation, an interaction term (days × case–control designation), and patient-specific and matched pair-specific random effects. Optimal glycemic control was defined as a mean glucose value less than 126 mg/dl during the year following diagnosis.

OS was defined as the time from lung cancer diagnosis until death from any cause. For OS, patients were considered censored at the last known date of life when death was not documented in the health records. PFS was defined as the time from lung cancer diagnosis until disease progression or death from any cause. Patients were considered censored at the last known date they were alive when disease progression or death had not occurred. Five-year OS was estimated with the Kaplan–Meier method and compared between subgroups by log-rank test. Variables with p < .10 were included in a Cox proportional hazards multivariable model to estimate the hazard ratio (HR) and 95% confidence interval (CI). In addition, a Cox proportional hazards regression was used to assess DM effect on OS and included matched pairs as the strata variable. Sample size was based on the number of available cases from 2007 to 2015; it provided 80% power to detect a difference in survival of 13% or higher at 5 years between the case group and controls group. p-values < .05 were considered statistically significant; SAS version 9.4 (SAS Institute, Inc.) was used for analysis.

## Results

### Patient characteristics

We performed chart reviews for 124 case–control pairs (248 patients). Table 1 summarizes patient characteristics. Mean age was 72 years, and most patients were white or from non-Hispanic race/ethnicity. The most common histologic factor was non-small-cell lung cancer, and almost 50% of patients had a diagnosis of stage IV disease. Most patients were married, consumed some alcohol, had a history of smoking, were retired and had a European Cooperative Oncology Group score of 1. DM patients had significantly greater BMI than patients without DM (p < 0.001).

**Table 1.** **Patient demographic characteristics by diabetes status.**

	**Presence of lung cancer and diabetes mellitus**			

**Characteristic^†^**	**No (n = 124)**	**Yes (n = 124)**	**Total (N = 248)**	**p-value**
Age, mean (SD), y	72.0 [8.7]	71.7 [8.6]	71.9 [8.6]	Matched

Male sex	81 [65.3]	81 [65.3]	162 [65.3]	Matched

White race/ethnicity	117 [94.4]	117 [94.4]	234 [94.4]	0.58

Tumor stage:				0.47

– Missing	20	22	42	

– I	26 [25.0]	27 [26.5]	53 [25.7]	

– II	4 [3.8]	4 [3.9]	8 [3.9]	

– III	18 [17.3]	28 [27.5]	46 [22.3]	

– IV	56 [53.8]	43 [42.2]	99 [48.1]	

Cancer type:				0.93

– Missing	0	3	3	

– NSCLC	104 [83.9]	101 [83.5]	205 [83.7]	

– SCLC	15 [12.1]	16 [13.2]	31 [12.7]	

– Other	5 [4.0]	4 [3.3]	9 [3.7]	

BMI, mean (SD), kg/m^2^	26.0 [4.7]	30.3 [6.4]	28.2 [6.0]	<.001

Married	99 [79.8]	86 [69.4]	185 [74.6]	0.22

Smoking status at time of cancer diagnosis:				0.43

– Never	16 [12.9]	12 [9.7]	28 [11.3]	

– Former	85 [68.5]	95 [76.6]	180 [72.6]	

– Current	22 [17.7]	17 [13.7]	39 [15.7]	

– Unknown	1 [0.8]	0 [0.0]	1 [0.4]	

ECOG PS score at time of cancer diagnosis:				

– Missing	1	3	4	

– 0	11 [8.9]	13 [10.7]	24 [9.8]	

– 1	86 [69.9]	71 [58.7]	157 [64.3]	

– 2	13 [10.6]	22 [18.2]	35 [14.3]	

– 3	11 [8.9]	13 [10.7]	24 [9.8]	

– 4	2 [1.6]	2 [1.7]	4 [1.6]	

^†^Values are presented as number and percentage of patients unless specified otherwise.

ECOG: European Cooperative Oncology Group; NSCLC: Non-small-cell lung cancer; PS: Performance status; SCLC: Small-cell lung cancer.

### Diabetes mellitus & lung cancer treatment characteristics

The mean [SD] duration of DM when lung cancer was diagnosed was 14 [11] years. The majority of patients were receiving oral agents at the time of their lung cancer diagnosis (Table 2). Only 15% of patients changed their DM therapy or began insulin therapy within a year of the cancer diagnosis, although data were not recorded for 24% of patients. DM complications were noted for only 9% of patients at the time of cancer diagnosis. Approximately, one half of the patients received chemotherapy. A wide variety of chemotherapy agents was used. A small percentage of patients (8%) were treated with targeted therapy and only two patients with immunotherapy. More than one half of patients received radiation at some point in their treatment. Among patients, 18 (15%) needed to change their DM therapy within 1 year of lung cancer diagnosis. One patient (6%) switched to diet control as DM therapy, six (33%) switched to oral treatment, ten (56%) switched to insulin, and one (6%) switched to oral treatment and insulin. Corticosteroids were taken by 44% of patients without DM and 52% of patients with DM.

**Table 2.** **Diabetes mellitus treatment of 124 patients with lung cancer.**

**Characteristic**	**Value^†^**
DM diagnosis preceded lung cancer diagnosis	113 [91.1]

Time since DM diagnosis if preceded cancer diagnosis, mean [SD], y	13.5 [11.1]

DM therapy	

Missing	3

Diet	22 [18.2]

Oral	65 [53.7]

Insulin	20 [16.5]

Oral + insulin	14 [11.6]

Insulin use at time of cancer diagnosis	

Missing	0

Yes	30 [24.2]

No	93 [75.0]

Unknown	1 [0.8]

^†^Values are presented as number and percentage of patients unless specified otherwise.

DM: Diabetes mellitus.

### Lung cancer effect on diabetes mellitus & metabolic control

**Figure 1.**
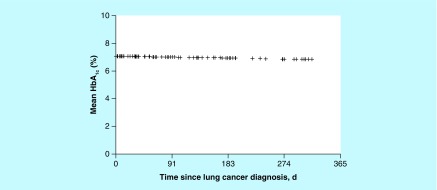
**Estimated mean hemoglobin A_1c_ value during the first year after lung cancer diagnosis for patients with diabetes mellitus.** HbA_1c_: Hemoglobin A_1c_.

The HbA_1c_ data measured within 1 year after lung cancer diagnosis were available for 51 patients with DM. Of these patients, 49% had at least one HbA_1c_ measurement of 7.0% or greater. Among those with DM, the mean HbA_1c_ within 1 year of cancer diagnosis was 7.0%. Mean HbA_1c_ among DM patients did not change significantly over time (p = 0.37) (Figure 1).

**Figure 2.**
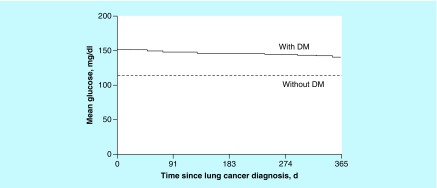
**Estimated mean glucose value during the first year after lung cancer diagnosis.** DM: Diabetes mellitus.

Glucose values during the year after lung cancer diagnosis were available for 118 and 119 patients with and without DM, respectively. Regarding DM, 112 patients (95%) had one or more glucose values of 126 mg/dl or greater, whereas only 83 patients without DM (70%) had these values. Mean (SD) glucose for the DM group was 149 (35) mg/dl; for the cohort without DM, mean (SD) glucose was 113 (17) mg/dl. Mean glucose level among patients with DM was significantly higher than patients without DM (p < 0.001) (Figure 2). Mean glucose values did not change significantly over time (0.130). Mean glucose level based on chemotherapy received and tumor progression was compared; no differences were noted between subgroups (p > 0.60).

### Diabetes mellitus effect on lung cancer survival

**Figure 3.**
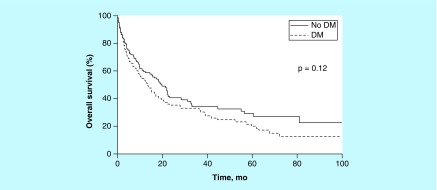
**Five-year overall survival stratified by diabetes mellitus status.** DM: Diabetes mellitus.

**Figure 4.**
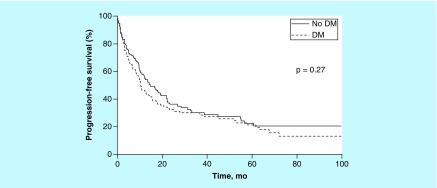
**Five-year progression-free survival stratified by diabetes mellitus status.** DM: Diabetes mellitus.

Median OS overall was 16.6 months (95% CI: 12.7–21.6 months); it was 13.3 months (95% CI: 9.9–20.0 months) for patients with DM and 19.5 months (95% CI: 14.6–28.5 months) for patients without DM (p = 0.12). Five-year OS was estimated at 20% for DM patients versus 29% for patients without DM, with a median follow-up of 26 months (Figure 3). The HR for matched pairs was 1.37 (95% CI: 0.91–2.02). Five-year PFS was estimated at 21% for DM cases versus 22% for non-DM cases (Figure 4). For this comparison, the HR for matched pairs was 1.33 (95% CI: 0.89–1.97). At 5 years, 13 patients with DM and 14 without DM were alive.

A borderline but not significant difference (p = .06) was observed for non-DM patients and DM patients with optimal glycemic control compared with DM patients without optimal glycemic control (19.5 months [95% CI: 15.3–23.4 months] vs 9.3 months [95% CI: 6.4–17.2 months]). The OS for DM patients based only on treatment (diet vs oral vs insulin ± oral) was compared, and no difference was seen (p = 0.52). However, the study's small numbers limit this comparison. In a multivariable model including sex, age, smoking status, type of cancer, DM and glycemic control, only smoking status and type of cancer were significant prognostic factors for OS (Table 3).

**Table 3.** **Multivariable analysis of overall survival.**

**Variable**	**HR (95% CI)**	**p-value**
Sex Male Female	1.0 (Reference) 0.82 [0.59–1.15]	.25

Smoking status Never Current Former	1.0 (Reference) 2.43 [1.13–5.23] 2.29 [1.15–4.57]	.02 .02

Age	1.02 [0.99–1.04]	.07

Type NSCLC SCLC Other	1.0 (Reference) 1.68 [1.09–2.60] 0.94 [0.32–2.73]	.02 .97

Diabetes mellitus No Yes	1.0 (Reference) 1.11 [0.76–1.60]	.60

Glycemic control No Yes	1.0 (Reference) 0.79 [0.54–1.15]	.22

CI: Confidence interval; HR: Hazard ratio; NSCLC: Non-small-cell lung cancer; SCLC: Small-cell lung cancer.

## Discussion

Little is known about how DM and solid-organ cancers interact to influence each other's outcomes on an individual level. We previously investigated effects of breast cancer and DM on patient outcomes and care [17], as well as these same effects of prostate cancer and DM [19]. In both analyses, DM did not impact survival in breast cancer or prostate cancer, and each cancer did not affect short-term (i.e., 1 year) glycemic control among patients with DM. We applied this methodology to study lung cancer, a non-sex-specific solid organ malignancy. Non-sex-specific malignancy such as lung cancer may interact differently in the presence of DM compared with malignancy such as breast or prostate cancer. A matched case–control analysis was used to investigate how DM affects lung cancer survival and how lung cancer affects metabolic control in DM.

In this study, we found that DM did not affect 5-year lung cancer OS or PFS. This outcome is congruent with our previous analysis [15], in which DM did not impact lung cancer survival negatively. The present study had many patient variables and a case–control approach – which were lacking in our previous analysis [15]. Furthermore, over 1 year, the various treatments for lung cancer did not impact negatively the metabolic control among lung cancer patients with concomitant DM.

Most patients in this study were elderly. This is important because less data are available in the literature regarding elderly patients with cancer compared with nonelderly patients with cancer. Although survival in recent years has improved for younger patients with cancer, older patients with cancer have not reaped such a benefit [21]. Our study adds to this area, which needs further research so efforts can be rendered to help rectify this disparity.

Our previous studies centered on two hormonally driven malignancies – prostate cancer and breast cancer. Since lung cancer is not hormonally driven, outcomes with respect to DM and lung cancer were expected to possibly be different. Cigarette smoking and tobacco exposure lead to increased insulin resistance and decreased effectiveness of insulin [22–24]. Such a milieu may contribute to altered inflammatory responses and may lead to an environment favorable to cancer cell survival and longevity. This result may lead to worse lung cancer outcomes and recalcitrant glycemic control. Furthermore, lung cancer is diagnosed at both a later age and a higher stage than prostate cancer and breast cancer. However, despite these differences, outcomes in our study with a nonhormonally driven cancer were similar to outcomes in our previous studies: DM did not affect lung cancer survival, and treatment of lung cancer did not affect glycemic control.

Five-year survival for lung cancer patients in these cohorts was far less than that seen in our previous studies involving patients with prostate cancer or breast cancer. This difference may be in part due to more lung cancer being diagnosed *de novo* at stage IV, unlike with the other cancers we have examined thus far. Screening for lung cancer has not yet been widely adopted. This lower survival rate in general for lung cancer is relevant for the findings of our study. The patients in cohorts of this study may not have lived long enough for opportunities to see change in glycemic control. It is also possible that progressive anorexia may have prevented any worsening of hyperglycemia.

In a meta-analysis, Zhu *et al*. [14] concluded that DM was associated with worse OS among lung cancer patients. However, the number of studies (20 cohort studies from 12 articles) in their meta-analysis was small. Hence, their conclusion is preliminary and needs to be corroborated in a prospective study or randomized trial, or both.

Data published by Arrieta *et al*. [25] showed that DM patients with proper glycemic control had a survival comparable with non-DM patients. In our study, we compared patient OS based on glycemic control (defined as mean glucose <126 mg/dl during 1 year following diagnosis). A borderline but not significant difference (p = .06) was observed for non-DM patients and DM patients with glycemic control compared with DM patients without glycemic control.

The present study has limitations. It was retrospective and some data were missing, despite meticulous data abstraction from direct chart review. The sample size was small, and the findings should be confirmed in a larger dataset and at other centers. The term of the study was short and should be corroborated over a longer period. In addition, the full cohort in this analysis was mostly white, and therefore results may have limited applicability to other race/ethnicity groups. Finally, official causes of death were not available for this study.

In conclusion, it appears that DM does not significantly impact survival in lung cancer patients adversely. In addition, lung cancer and its treatment do not appear to affect glycemic control. These data add to our publications about the interplay between DM and cancer on an individual level.

## Future perspective

With the findings of this study, providers can be reassured that DM does not affect lung cancer OS and PFS, and that treatment of lung cancer does not negatively affect glycemic control among patients with DM. Future continued study is needed to address optimal care for patients with these concomitant diagnoses.

**Summary points**

- Impact of lung cancer or its treatment on diabetes mellitus (DM) and the impact of DM on lung cancer survival are unknown on an individual level.
- DM patients had a significantly higher BMI (p < .001).
- Significant differences in glucose within 1 year of cancer diagnosis were seen between groups in sex and in use of corticosteroids, chemotherapy, targeted therapy and radiation therapy (all p < .03).
- Among patients with DM, the mean hemoglobin A_1c_ was 7.0% within 1 year of cancer diagnosis.
- In Kaplan–Meier survival analysis (median follow-up: 26 months), 5-year overall survival was estimated at 20% for DM patients versus 29% for patients without DM. The hazard ratio for matched pairs was 1.37 (95% confidence interval: 0.91–2.02).
- Five-year progression-free survival was estimated at 21% for DM patients versus 22% for patients without DM. The hazard ratio for matched pairs was 1.33 (95% confidence interval: 0.89–1.97).
- Mean hemoglobin A_1c_ and glucose values among DM cases did not change significantly over time.
- Mean glucose level among DM patients was significantly higher than for patients without DM (p < .001).
